# Clinical Research Nurses, an Untapped Resource of Nurse Researchers?

**DOI:** 10.1002/nop2.70524

**Published:** 2026-04-29

**Authors:** Faye Forsyth, Miriam Avery, Sandra Bartolomeu Pires, Chantelle Moorbey, Layla Bolton Saghdaoui, Amanda Skinner, Vittoria Sorice, Mary Harrison, Rosalynn C. Austin

**Affiliations:** ^1^ PACE Section(Perioperative, Acute, Critical Care and Emergency Medicine), Department of Medicine University of Cambridge Cambridge UK; ^2^ KU Leuven Department of Public Health and Primary Care Leuven KU Belgium; ^3^ University Hospital Southampton NHS Foundation Trust Southampton UK; ^4^ University of Southampton School of Health Science Southampton UK; ^5^ NIHR ACR Wessex Southampton UK; ^6^ NIHR South Central RRDN Southampton UK; ^7^ Imperial College Healthcare NHS Trust London UK; ^8^ Imperial College London London UK; ^9^ South West NHS Genomic Medicine Service Exeter UK; ^10^ Chesterfield Royal Hospital NHS Foundation Trust Chesterfeild UK; ^11^ University of Derby Derby UK; ^12^ University Hospitals of Leicester NHS Trust Leicester UK; ^13^ University of Leicester Leicester UK; ^14^ Portsmouth Hospitals University NHS Trust Portsmouth UK

## Abstract

The clinical research nurse (CRN) workforce in the United Kingdom (UK) is considerable and has a level of expertise when it comes to delivering research in a healthcare setting. Yet these same individuals are often overlooked when it comes to developing future academic nursing research leaders within the UK. A group of current or former clinical research nurses (the authors), held a reflective discussion and asked themselves: “Are CRN's a potential untapped resource who could plug the gap in the academic researcher workforce?” This brief report presents our reflections from personal experiences, augmented by published evidence, to open a dialogue on the potential role of CRNs as academic nurse researchers. We concluded that while CRNs have the necessary aspirations, skills and attributes, there remain key barriers which require this group of nurses to rely on personal determination and persistence to become academic nurse researchers.

## 1 Introduction

The clinical research nursing workforce in the United Kingdom (UK) has grown substantially over the past decade. A recent UK wide scoping census suggests the number of Clinical Research Nurses (CRNs) is as high as 7469 (Whitehouse C et al. 2023) we estimated they represent 0.8% of the nursing workforce registered on the Nursing and Midwifery Council (NMC) register.^1^ (Nursing and Midwifery Council 2023). As a percentage, this may seem like a small, even inconsequential number. However, when put into the context of other sub‐specialities like advanced practitioners (~2% of the nursing workforce) (Palmer William and Vaughan 2023) and midwives (~5.3% of the NMC register) (Nursing and Midwifery Council 2023), it is clear the clinical research nurse workforce is substantial.

Whilst CRNs play a large part in delivering research that underpins evidence‐based practice, they do not frequently set the research agenda. Nurse‐led research is typically driven by academic researchers and clinical academics who sit within higher education institutions. Following their 2019 census of the academic workforce in health faculties, (Council of Deans 2019) the UK Council of Deans of Health highlighted concerns about the capacity and capability of the current academic workforce, citing recruitment and retention challenges, the advancing age of those currently in post and a lack of diversity. Notably, only a small percentage of those with a research remit possessed a doctorate (28%) (Council of Deans 2019). Whilst this might be unique to the UK higher education system, it is nevertheless concerning given the need to build a stronger and appropriately qualified academic nurse workforce who will support future aspiring nurse researchers.

## 2 Background

In the last 4 years in the UK, the Chief Nursing Officer (CNO) and the National Institute for Health and Care Research (NIHR) have launched campaigns to encourage more nurses and midwives to embark on the pathway to becoming a nurse researcher. Both the CNO and NIHR released strategic and implementation programmes to deliver on these aspirations (National Institute for Health and Care Research 2019, 2023; NHS England and NHS Improvement 2022). These campaigns included developing more diverse, fair and embedded structures to facilitate nurses in any role and setting to pursue clinical academic careers, which theoretically includes CRNs.

Despite the drive to get more nurses into research roles, audits show that nurses remain grossly underrepresented—only 0.1% of research active staff are nurses, compared to 4.6% of medical staff (Church 2023). Further, it is becoming evident that supporting aspiring nurse academics through doctoral training programmes is just the tip of the iceberg (Negarandeh and Khoshkesht 2022). Indeed, the NIHR recognises the need for clearer pathways and more robust career development support to enable PhD qualified nurses to become independent nurse researchers (Church 2023). What remains less certain is whether these NIHR doctoral training initiatives and post‐doctoral career development pathways are open and attuned to the specific needs of CRNs. For whilst CRNs will develop advanced clinical and research skills, they perceive themselves to be ‘invisible’ in the milieu of healthcare professionals (Kunhunny and Salmon 2017). Further, their mapped career development pathways do not include academic training pathways; rather, research management and leadership roles (Kenkre and Foxcroft 2001; Ward and Dorgan 2024).

## 3 Context

Given the tension between the need for more doctoral qualified academic nurse researchers and the apparent oversight of a very able faction of the nursing workforce for these roles, the first and corresponding authors (FF and RCA) contemplated: “Are CRNs a potential untapped resource who could plug the gap in the academic researcher workforce?” After our initial reflective discussion, we approached CRN colleagues to informally explore their perspectives on CRNs as future academic nurse researchers. In addition to asking them the question we asked ourselves, we asked them to share their personal reflections on barriers and facilitators they might have experienced while developing skills as academic nurse researchers. We accessed our networks and their networks to identify fellow CRNs who were attempting to grow careers as nurse academics/researchers. Nine current or former CRNs, predominantly from the south of England, from varying specialities and with differing levels of experience, provided feedback. All participating colleagues were asked the same questions via email (Box 1) and agreed to contribute to the summarisation of the collated feedback as co‐authors.^2^

Box 1: Questions Asked to Co‐Authors

1. What led you from clinical research nurse to academic nurse researcher? Or what has prohibited you from becoming an academic nurse researcher whilst a clinical research nurse?
2. What enabling factors can you describe?
3. Please describe three skills or abilities that clinical research nursing has equipped you with that have subsequently made your transition to academic researcher easier.
4. Please describe three clinical research nurse related factors that have made your transition to an academic nursing role more difficult.

Feedback from all authors was collated (within NVivo software, used only to organise co‐author inputs) and inspected for similarities across the narratives. Reflective narratives were integrated with published evidence to produce a brief report. It is important to state that, whilst we have employed some principles of academic research, this brief report is not intended to be conveyed, nor interpreted as empirical research. Rather it is our collective personal experiences of pursuing academic nurse researcher careers whilst being employed as CRNs, or with backgrounds in clinical research nursing. We use these experiences to open a dialogue in the nursing community about CRNs as future academic nurse researchers.

## 4 Discussion

We posit that CRNs may have the necessary aspirations, skills, and attributes to contribute to the long‐term sustainability of the academic nurse researcher workforce. The following sections shine a spotlight on the existing research evidence and are integrated with our personal accounts as a way of illustrating how we came to our conclusions on the ‘fit’ of the CRN as potential academic nurse researchers.

**CRNs aspire to develop into academic nurse researchers—**our aspiration to become nurse researchers developed at varying stages of our careers, spanning from our days as student nurses to our roles as seasoned healthcare professionals. Regardless of when and how, we all consciously aspired to follow this path.

I felt I could do more. My brain was wired to do more. Nurse 1

I really enjoyed the research dissertation element of my undergraduate degree from then on, I wanted to be a nurse researcher. Nurse 3

We are not alone in our aspirations. A recent cross‐sectional survey of ~300 nurses involved in research delivery at nine sites in NHS England, reported that 22% already aspired to be research leaders, while 45% indicated they had future aspirations for such a career (Avery and Griffiths 2023).

**CRNs possess multiple research skills**—CRNs cultivate a deep understanding of research delivery, equipping them with the necessary knowledge and skills to take a fledgling project from conception to fruition with relative ease. Being experienced in working closely with patients, assessing feasibility, obtaining regulatory approvals, and the process of systematically implementing protocols has distinct advantages when undertaking the development and coordination of their own research endeavours. Further, constant exposure to, and collaboration with research professionals, enhances communication and networking skills. Equally, being at the coalface of research delivery equips CRNs with an acute appreciation of, and accountability to, participants' research experiences. Such insights can pay dividends when designing studies (Shiely et al. 2023), and communicating research findings. Our experiences as emerging nurse researchers matched literature‐based observations.

My early training and experience in research delivery gave me a huge advantage once I came to think about developing research ideas. I had a sense of what was realistic and deliverable. It also enabled me to understand the mandatory logistics that are needed like local approvals and ethics. Nurse 2

I had to field the disappointment in a participant when, on their last follow‐up visit, I had nothing to offer them in terms of what their participation meant to the researchers. This has turned me into a nurse researcher who is passionate about transparent dissemination for all. Nurse 4

While these delivery skills transferred well for the aspiring academic nurse researcher, they are not in themselves sufficient to ensure success. We identified two core attributes that were required to make the transition from CRN to academic nurse researcher.

**CRNs have the key attributes required to develop as academic nurse researchers**—Curiosity and tenacity were described as pivotal characteristics within our group that prompted and sustained a transition from CRN to academic nurse researcher. A curiosity to know more, to ask questions beyond the research being implemented and a desire to bring a ‘nursing lens’ to the problems observed in practice sparked many of our nurse researcher journeys. However, to sustain the pursuit of the nurse researcher career, we shared stories of the need to have considerable tenacity and resilience.

The transition from research nurse to nurse researcher was not a decision I made lightly. It was driven by a deep passion for creating new knowledge and wanting to have a more direct influence on research outcomes. Nurse 6

They have to really want it—it's tough so not to be undertaken on a whim, they need to be resilient, if they give up at the first hurdle, they're unlikely to get far as a researcher. Nurse 8

There were times when I questioned whether the sacrifice was worth it, but my passion for research and my desire to make a meaningful impact kept me motivated. Nurse 7

Barriers, beyond those we experienced, are already established and plentiful (Avery et al. 2022; Trusson et al. 2019). Making the leap means accepting a certain amount of personal risk and being prepared to go the extra mile.

Having identified the aptness of CRNs as future nurse researchers, it is imperative to highlight to strategic leaders and the nursing community the barriers, facilitators, and possible solutions to realising that potential.

**Barriers to a CRN's journey to becoming an academic nurse researcher**—Several barriers were consistently voiced by our contributors at all points of the trajectory (i.e., from pre to post‐doctoral stages) which aligned with published barriers (Avery et al. 2022). The alignment of barriers included: limited funding availability for nurses; difficulties in finding appropriate nursing supervisors; and rigid organisational structures which prohibited dual clinical/academic roles. Multiple studies have already highlighted inequalities within the healthcare workforce in terms of funding and opportunities, with nurses notably under represented (Henshall et al. 2021). Once aspiring academics have completed post‐graduate degrees, similar obstacles appear to be reported by all staff groups (Trusson et al. 2019). Novel barriers identified by the authors who have all had roles as CRNs, included location (rurality may play a role in accessing opportunities) and the absence of certain nursing specialisms (e.g., research delivery).

Not being linked to a clinical disease area, therefore my PhD and work life are separate. I imagine it would be easier if my PhD related to my work! Nurse 8

Having access to compare the activity across what was 11 and now 9 Trusts (2 mergers), I can see that living near a tertiary centre or a teaching hospital gives more opportunity to nurses to become nurse researchers. Nurse 5

**Facilitators to a CRN's journey to becoming academic nurse researchers**—Key facilitators to embarking on and/or completing the authors' academic journeys were support and mentorship. Support is required at multiple levels, locally from colleagues and managers, and organisationally via institutions and funding schemes. While accessing support may be challenging for CRNs, for those who do connect with the right people, the impact is beneficial.

Although I was enthusiastic, I was lucky enough to be in a department that supported me to be creative and think about how I could contribute to research beyond recruitment and collecting data. Nurse 2

Key mentors around me (consultant [physician] and nursing leaders) pushed me to apply for a PhD fellowship and now I'm an early career nurse researcher. Nurse 4

Having managers ‘on your side’ is key. Applications will ask for departmental commitment and letters of support, and boards/committees will see through these if they aren't genuine promises of support. Nurse 1

## 5 Final Thoughts

CRNs can manifest the necessary aspirations, skills, and attributes to develop into academic nurse researchers. They are uniquely situated to supplement the dwindling numbers of nurse researchers within UK higher education institutions. Shortcomings in infrastructure, funding schemes, and future career opportunities mean that CRNs struggle to make the transition. Nursing leaders with a mandate to increase the nurse academic and research workforce should make changes (Box 2) to enable CRNs with aspirations to develop a career as an academic nurse researcher. The current reliance on personal tenacity and luck in terms of infrastructural support and mentorship is unsustainable and prohibits this promising source of future academics being leveraged.

Box 2: Impact statement, suggested actions and trackable outcomes.

Managers:

- Apply the chief nursing officer for England's strategic plan for research (May 2021).
  - Report nurse research activity to national nursing leadership.
- Paired mentorship with nurse academic principal investigators.
  - Track number of mentorship pairs.

National Nursing Leadership:

- Pilot bridging fellowships, internships and other support mechanisms and pathways.
  - Collect success metrics to determine what ‘good looks like.’
- Ensure research‐focussed non‐executive directors (who are nurses) are incorporated on every NHS Board to champion and protect nurse research (Waters 2026).
  - Report the presence or absence of a board member who is a senior nurse with a strong academic and research track record.
- Supported career pathways within CRN careers and banding structure for the transition to academic nurse researchers.
  - National NHS adaptation of CRN career structure and banding to be adopted.

Funders:

- Clear eligibility criteria for CRN's within NIHR programs.
  - Updated wording and inclusiveness to be published by NIHR.
  - Collect metrics on CRN fellowship and funding applications.

It is crucial to acknowledge that not all CRNs will aspire to become nurse researchers, and the speciality has immense value (Hansen et al. 2022). On behalf of those CRNs in the UK who seek to pursue the development of research programmes, we ask senior research leaders and funding bodies to consider the evidence and our experiences described in this brief report. As many within our writing group demonstrate, CRNs possess the requirements to make great academic nurse researchers and their potential to supplement the waning pool should not be overlooked.

## Author Contributions

R.C.A. and F.F. conceived of and drafted the article. All other authors were asked to comment on their experiences as clinical research nurses seeking to become nurse researchers. Quotes used in this manuscript come from the authors' personal reflections related to their experiences as clinical research nurses who, to varying degrees, have worked as academic nurse researchers. All authors have edited and approved the final manuscript.

## Funding

The authors have nothing to report.

## Disclosure

The authors of this editorial have all been clinical research nurses. At the time of article submission (2024), only three of the nine have remained as CRNs, one of which was on a secondment from that role to enable further development as a Nurse Researcher. To pursue their goals, three have left being a CRN, moving to more academic or ACP roles to facilitate the goal of becoming nurse researchers. The remaining three had a change in circumstances that led to them leaving their roles as CRN. This editorial is a combination of their opinions and experiences, not the opinion of any of the institutions to whom they are affiliated. All quotes in this article were provided by the authors and they give their permission for publication.

At the time of conceptualization and writing, R.C.A. was employed by the University of Stavanger in Norway, where she received funding from the Norwegian Research Council (Grant No. 301472) as a part of her role as a post‐doctoral researcher for the eHealth @Hospital to Home project at the University of Stavanger. R.A. receives support from the National Institute of Health and Care Research (NIHR) Applied Research Collaboration Wessex through a “Pre‐Application Support Fund” post‐doctoral award. R.C.A. was supported by the National Institute for Health and Care Research (NIHR) Applied Research Collaboration (ARC) Wessex. F.F. was employed as Lead Research Nurse of the OPTIMISE HFpEF study and was registered as a PhD student at the University of Cambridge. F.F. received funding from the Evelyn Trust (Grant number 21/15).

The research time of L.B.S. (grant number NIHR302917) is funded by the National Institute for Health and Care Research (NIHR). C.M. PhD training and time is supported by Novo Nordisk UK Research Foundation. M.E.H. is supported by the Wellcome Trust as part of the Leicestershire Health Inequalities Improvement Programme [223512/Z/21/Z]. M.A. holds a “Development and Skills Enhancement Award” (NIHR305096) and her research time is funded by the NIHR.

The views expressed in this publication are those of the author(s) and not necessarily those of the NIHR, NHS or the UK Department of Health and Social Care.

## Ethics Statement

The authors have nothing to report. All quotes were obtained from the authors in discussing their experiences as clinical research nurses about their experiences as new nurse researchers.

## Consent

The authors have nothing to report.

## Conflicts of Interest

The authors declare no conflicts of interest.

## Data Availability

Data sharing not applicable to this article as no datasets were generated or analysed during the current study.

## Endnotes

^1^
The percentage presented in the text (0.8%) was based on the following formula: (*n* = clinical researcher nurses responding to last census) [/divided by] (*n* = nurses registered with NMC in England) [× multiplied by 100] = % (percentage of research nurses per number of registered nurses). Whitehouse et al. state CRNs represent 2% of the NHS workforce. The calculation performed was based on NHS England workforce statistics from April 2022 (*n* = 382,566). However, as the report details the total number of CRNs across the UK (e.g., Scotland, England, Northern Ireland and Wales), this figure may be artificially high. When recalculated based on the England only census figures (CR Nurses *n* = 5973; CR Midwives *n* = 353; total *n* = 6326), the proportion of the workforce in England self‐identifying as CRNs based on the 2022 figures presented is 1.6%. We have calculated that if the number of CRNs has remained stable since 2022, they represent 0.8% of the nursing and midwifery workforce registered with the NMC in 2024 [*n* = 809176].

^2^
This is not a piece of research work; it is a comment piece that illustrates the personal experiences of the authors augmented by current literature. Our experiences are from multiple health trusts across the UK with the majority located in the south coast of England. It is a relevant and current issue actively being debated within the UK. Further research would be required to confirm our perspectives on the issue of CRN being offered more supportive pathways for with ambitions to pursue a clinical academic career.
